# Previous exercise training increases levels of PPAR-α in long-term post-myocardial infarction in rats, which is correlated with better inflammatory response

**DOI:** 10.6061/clinics/2016(03)08

**Published:** 2016-03

**Authors:** Marília Harumi Higuchi Santos, Maria de Lourdes Higuchi, Paulo J F Tucci, Shérrira M Garavelo, Márcia M Reis, Ednei L Antonio, Andrey J Serra, Raul Cavalcante Maranhão

**Affiliations:** I Hospital das Clínicas da Faculdade de Medicina da Universidade de São Paulo, Instituto do Coração (InCor), Laboratório de Patologia Cardíaca, São Paulo/, SP, Brazil; II Universidade Federal de São Paulo (UNIFESP), Cardiologia; III Fisiologia Cardíaca, São Paulo/, SP, Brazil; IV Universidade Nove Julho, Programa de Pós Graduação em Biofotônica Aplicada às Ciências da Saúde, São Paulo/, SP, Brazil

**Keywords:** Exercise Training, Myocardial Infarction, PPAR-α, Apoptosis, Inflammation

## Abstract

**OBJECTIVE:**

Exercise is a protective factor for cardiovascular morbidity and mortality, with unclear mechanisms. Changing the myocardial metabolism causes harmful consequences for heart function and exercise contributes to metabolic adjustment modulation. Peroxisome proliferator-activated receptors (PPARs) are also myocardium metabolism regulators capable of decreasing the inflammatory response. We hypothesized that PPAR-α is involved in the beneficial effects of previous exercise on myocardial infarction (MI) and cardiac function, changing the expression of metabolic and inflammatory response regulators and reducing myocardial apoptosis, which partially explains the better outcome.

**METHODS AND RESULTS:**

Exercised rats engaged in swimming sessions for 60 min/day, 5 days/week, for 8 weeks. Both the exercised rats and sedentary rats were randomized to MI surgery and followed for 1 week (EI1 or SI1) or 4 weeks (EI4 or SI4) of healing or to sham groups. Echocardiography was employed to detect left ventricular function and the infarct size. Additionally, the TUNEL technique was used to assess apoptosis and immunohistochemistry was used to quantitatively analyze the PPAR-α, TNF-α and NF-κB antigens in the infarcted and non-infarcted myocardium. MI-related mortality was higher in SI4 than in EI4 (25% *vs* 12%), without a difference in MI size. SI4 exhibited a lower shortening fraction than EI4 did (24% *vs* 35%) and a higher apoptosis/area rate (3.97±0.61 *vs* 1.90±1.82) in infarcted areas (both *p*=0.001). Immunohistochemistry also revealed higher TNF-α levels in SI1 than in EI1 (9.59 *vs* 4.09, *p*<0.001) in infarcted areas. In non-infarcted areas, EI4 showed higher levels of TNF-α and positive correlations between PPAR-α and NF-κB (r=0.75, *p*=0.02), in contrast to SI4 (r=0.05, *p=*0.87).

**CONCLUSION:**

Previously exercised animals had better long-term ventricular function post-MI, in addition to lower levels of local inflammatory markers and less myocardial apoptosis, which seemed to be related to the presence of PPAR-α.

## INTRODUCTION

Several epidemiological studies have shown that exercise not only diminishes the incidence of myocardial infarction (MI) but also attenuates the complications and the mortality rate of MI 1,2. Experimental studies specifically suggest that exercise training prior to MI protects against the pathological remodeling and ventricular dysfunction induced by MI 3,4,5. Moreover, rats submitted to regular exercise training before induction of MI by coronary artery ligation presented a reduced MI size, together with improvement of myocardial vascularization and function 6.

The effects of previous exercise training on myocardial metabolism after MI have been studied. Hypoxia dramatically changes this metabolism by switching the preferential energy source from fatty acid oxidation to glucose consumption 7. These metabolic changes may lead to harmful consequences for heart function in the long term 8, but exercise can contribute to modulating such post-MI metabolic adjustment.

Peroxisome proliferator-activated receptors (PPARs) are involved in mediating numerous physiological effects in humans, including glucose and lipid metabolism. PPAR-α ligands effectively treat dyslipidemia and have significant anti-inflammatory and anti-atherosclerotic activities 9. Apart from the ligand recognition involved in overall cardiac function, these ligands have additional beneficial functions, such as regulating interactions with co-factors involved in signal transduction during transcription and binding to homodimerization or heterodimerization partners 10,11. In particular, PPAR agonists are key regulators of myocardial metabolism that are known to decrease the infarct size and the inflammatory response in experimental MI 12.

PPAR-γ co-activator 1-alpha (PGC-1α) is currently considered to be a major regulator of phenotypic adaptation induced by exercise 13 and has been identified as a transcriptional co-activator of PPARs 14.

The nuclear transcription factor NF-κB stimulates cytokine expression in the myocardium 15 and has inflammation-enhancing action as well as being associated with the development of apoptosis. It has been found that fenofibrate (an agonist of PPAR-α) inhibits cardiac hypertrophy by negatively regulating the activation of NF-κB 16.

The aim of the present study was to test the hypothesis that exercise-trained animals with long-term post-MI have better ventricular function than non-trained animals do, accompanied by PPAR-α activation. This activation may in turn decrease inflammatory response regulators, such as TNF-α and NF-κB, reducing apoptosis in the myocardial tissue.

## METHODS

### Experimental design

Female Wistar rats weighing 250 to 290 g were assigned to exercised or sedentary groups. The exercised rats were submitted to daily swimming sessions five days/week for eight weeks. Initially, adaptation to training exercise was performed for one week; the first session lasted for 15 min and the next sessions had their duration increased by 15 min each, reaching 60 min on the fourth day, which was maintained until the end of training. After eight weeks, the animals were randomized into non-infarcted (sham) or infarcted groups. After MI induction in the latter groups, the rats were followed for 1 or 4 weeks of healing with no additional exercise and they were then sacrificed to evaluate changes in the damaged tissue. The animals were subdivided into the following groups: sham, exercised (ESh, n=10) or sedentary (SSh, n=10) and sacrificed after 4 weeks; infarcted, exercised (EI1, n=8) or sedentary (SI1, n=6) and sacrificed after 1 week; or infarcted, exercised (EI4, n=12) or sedentary (SI4, n=12) and sacrificed after 4 weeks.

At the end of the experiment, the rats were sacrificed by urethane overdose (4.8 g/kg intraperitoneally). After sacrifice, the rats' hearts were harvested in 10% buffered formalin for analysis.

The experiment was performed in agreement with the *Guide for the Care and Use of Laboratory Animals* published by the US National Institutes of Health (NIH publication no. 85-23, revised 1996) and the policy described in *The Journal of Physiology* 17.

### MI induction

Rats had their left anterior descending coronary artery anesthetized (intramuscular xylazine, 10 mg/kg, and ketamine, 90 mg/kg), ventilated with Harvard 683 respirator(Harvard Apparatus, Holliston, Massachusetts, USA), 2.5 ml, 75 to 78 strikes/min and permanently ligated with a 5/0 silk thread.

### Echocardiography and infarct size

Transthoracic echocardiography was performed 48 h after MI induction or sham operation using a 12-MHz transducer (Sonos-5500, Phillips). M-mode tracing of the left ventricle (LV) was obtained from the parasternal long-axis view to measure the left ventricular end-diastolic diameter (LVEDD) and the left ventricular end-systolic diameter (LVESD) and the shortening fraction (SF) (%) was calculated. An estimate of the infarct size was obtained as the percentage of dyskinetic wall in relation to the internal LV perimeter. Bi-dimensional images of the LV at basal, mean and apex transversal axes were specifically obtained to determine the infarct size. Along each axis, the internal perimeter of the ventricular cavity was measured in diastole, as was the length of the arc corresponding to the infarcted region. For each axis, we calculated the infarct size as the mean number of the three measures obtained. MI was considered to be present when echocardiography images showed higher echogenicity and/or changes in the thickness and systolic movement of the myocardium (akinesia or dyskinesia).

### Immunohistochemistry

After sacrifice, the animals had their hearts harvested with 10% buffered formalin and 2 cm myocardial sections from the medial poles of the resected fragments were processed for embedding in paraffin. The material was submitted to serial 5 µm sections for staining with hematoxylin and eosin and for immunohistochemistry using immunoperoxidase and the following anti-human primary antibodies: anti-PPAR-α (C-20) goat polyclonal (Santa Cruz Biotechnology), rabbit anti-TNF-α polyclonal (Abcam), and rabbit anti-NF-κB p65 polyclonal (Abcam) antibodies.

Antigen retrieval was performed in a pressure cooker by heating the sections with citrate buffer, followed by recovery with 6% hydrogen peroxide to block endogenous peroxidases. The sections were then incubated with a serum-free protein block (Carpinteria, CA, USA) to inhibit non-specific binding. For the specimens that were treated with a specific monoclonal primary antibody, a biotin-labeled rabbit anti-mouse anti-serum was used as a secondary antibody (Dako, Glostrup, Denmark), followed by a streptavidin- horseradish peroxidase complex label (Amersham, Little Chalfont, UK) diluted 1:200 in phosphate-buffered saline (PBS). The staining procedure was completed using diaminobenzidine (DAB; Sigma Chemical Corporation, St. Louis, MO) as a chromogen. For the specimens that were treated with a polyclonal primary antibody, a goat secondary antibody was used. Finally, the sections were counterstained with Harris' hematoxylin. A reaction without the primary antibody was performed as a negative control.

### Antigen quantification

**Figure 1:**
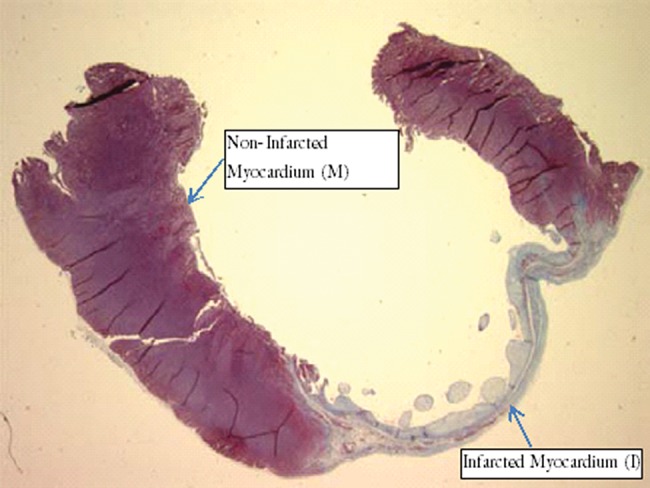
Myocardium (M) and Infarction (I) from animal of sedentary group. Magnification, 0.8x.

The positive percentage area for each antigen in the infarcted myocardium (I) and non-infarcted myocardium (M) regions was detected using an Aperio ImageScope automatic color detector analysis system, as shown in Figure 1. Delimited areas selected for antigen quantification were used for comparison of the I and M region sizes.

### Apoptosis detection

Paraffin-embedded, 5 µm-thick myocardial sections were submitted to the fluorescent TUNEL technique to detect apoptotic cells. More specifically, these sections were de-waxed in xylene and rehydrated and were then pretreated with proteinase K (Gibco, Gaithersburg, MD) (20 µg/mL) diluted in 10 mM Tris-HCl, pH 7.5, for 15 min at 32–35°C; rinsed in 0.1 M PBS, pH 7.4; and incubated in a humid atmosphere at 37°C for 60 min in 50 µL of TUNEL reaction mixture coupled with fluorescein (Boehringer, Mannheim, Germany). Sections of myocardium from patients with Chagas' disease presenting high numbers of apoptotic interstitial cells were used as a positive control, and the terminal transferase component was omitted from the mixture as a negative control. TUNEL-stained sections were analyzed under a fluorescence microscope by two independent researchers (MS and RI). Positive cells were counted in all fields each case I and M regions at 20x magnification.

### Statistical analysis

All values are represented as mean numbers. To compare variables in equivalent regions between the exercised and sedentary infarcted groups under similar clinical conditions (1 week or 4 weeks post-MI), we used Student's t-tests or Mann-Whitney tests, depending on the normal or non-normal distribution of the data. We used Kruskal-Wallis one-way analysis of variance (ANOVA) on ranks to compare all groups and differences were detected using all pairwise multiple comparison procedures (Bonferroni t-test) if the distribution was normal and Dunn's method when the distribution was not normal. Simultaneous comparison of 6 groups, including sham groups, led to many situations not being tested. Correlations between the studied variables were performed using the Spearman correlation test. Probabilities of <0.05 were considered statistically significant. All of the statistical tests were performed by SigmaStat 3.5 statistical software.

## ETHICS

This study was approved by the Ethics Committee of the Universidade Federal de São Paulo (UNIFESP).

## RESULTS

### Mortality

Postoperative mortality was significantly higher in the SI4 group than in EI4 (25% *vs* 12%, *p*<0.05). No animal deaths were registered beyond the first 24 h.

### Echocardiographic data

The infarct size estimated by echocardiographic measurement was 35% of the internal perimeter of the LV in SI4 and 41% in EI4. LVESD and LVEDD measures did not differ between groups, but SI4 exhibited a lower SF compared with EI4, indicating poorer LV function/contraction in SI4 (Table 1).

### Immunohistochemistry

**Figure 2:**
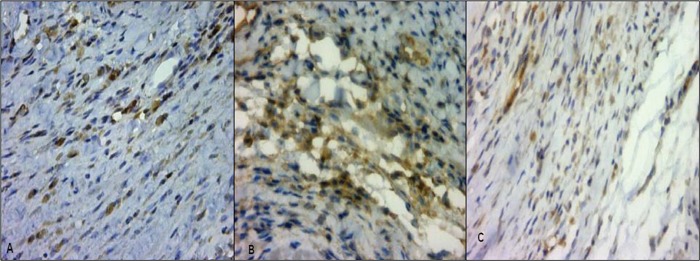
Site of infarction, stained by immunohistochemistry, with brown staining indicating PPAR-α (A), NF-κB (B) and TNF-α (C). Original magnification, 40x.

Antigen detection was performed by assaying differences in brown staining (Figure 2). The mean positive percentage areas for PPAR-α, TNF-α and NF-κB in the I and M regions in the different groups are shown in Table 2. The infarcted area delimited for antigen quantification was 7.12 mm^2^ in SI4 and 7.26 mm^2^ in EI4, with no significant difference (*p*=0.90).

### Early post-MI changes

The positive percentage areas for the antigens are shown in Table 2. SI1 exhibited higher levels of TNF-α than EI1 did at in the myocardial infarct and the myocardium distant from the infarct (both *p*<0.05). Comparison of the other antigens could not be performed or was not statistically significant. Despite no significant difference in PPAR-α and NF-κB levels, there were strong positive correlations between PPAR-α and TNF-α (r=0.929, *p*<0.001) and PPAR-α and NF-κB (r=0.786, *p*=0.01) in EI1, but not in SI1 (r=0.086, *p*=0.92 and r=-0.029, *p*=1, respectively). Between TNF-α and NF-κB, there was a significant positive correlation in EI1 (r=0.69, *p*=0.05) and a negative correlation in SI1 (r=-0.943, *p*=0.02).

### Long-term post-MI changes

The mean percentages of area positive for PPAR-α, TNF-α and NF-κB after 4 weeks are shown in Table 2. In MI areas, there were higher amounts of PPAR-α (*p*=0.04) and NF-κB (*p*=0.02) than in SI4, and there was a positive correlation between these two antigens in EI4 (r=0.750, *p*=0.02), but not in SI4 (r=0.18, *p*=0.66).

### Sham groups

There was no significant difference in PPAR-α, NF-κB or TNF-α between the sham sedentary and exercised groups. However, there was a positive correlation between PPAR-α and NF-κB in ESh (r=0.71, *p*=0.02), but not in SSh (r=-0.224, *p*=0.51).

### Comparison of early and long-term changes

Comparing the sedentary and exercised groups 1 and 4 weeks post-MI using ANOVA on ranks, we noted that after 4 weeks of healing post-IM, both the sedentary and the exercised groups presented significantly lower levels of NF-κB in the healthy myocardium (SI1xSI4 *p*<0.001; EI1xEI4, *p*<0.001). There were also decreased levels of PPAR-α in the sedentary group after four weeks (SI1xSI4, *p*=0.03), but no difference in the exercised group (EI1xEI4, *p*=0.08). Most of the variables decreased significantly after 4 weeks compared with 1 week. In the infarction area, the exercised group also presented decreased levels of NF-κB after 4 weeks (EI1xEI4, *p*<0.05).

### Apoptosis

**Figure 3:**
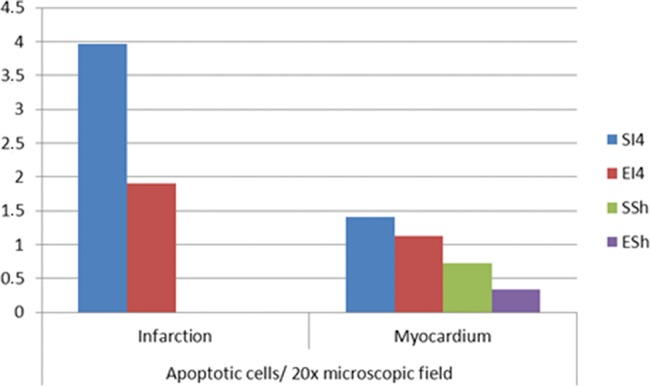
Number of apoptotic cells per field in the infarcted and non-infarcted myocardium.

The mean number of apoptotic cells per field at 20x magnification was higher in SI4 than in EI4 in the I region (3.97±0.61 *vs* 1.90±1.82, *p*<0.01) and in the M region (1.41±0.57 *vs* 1.13±0.64). In the SSh and ESh groups, the mean numbers of apoptotic cells per field in the M region were 0.73±0.61 and 0.34±0.19, respectively. In SI4, there was a higher apoptosis rate in the I region compared with the M region (*p*<0.001). However, in EI4, the numbers of apoptotic cells per field did not differ between the studied regions. In the I region in particular, SI4 exhibited a higher apoptosis rate compared with EI4 (*p*<0.05) (Figure 3).

## DISCUSSION

Two of the key determinants of MI outcomes are the inflammatory and pro-apoptotic responses triggered after MI occurrence 18,19. These responses facilitate survival during the acute phase but can be harmful in the long term. It is known that exercise training decreases chronic inflammation 20 and enhances innate immune function 21.

Several studies have shown that gender can be a key factor in cardiac remodeling post-MI and could be a risk factor for an unfavorable prognosis 22,23. However, a study comparing male and female rats 6 weeks after MI showed no gender differences in morphological and functional abnormalities or in LV remodeling post-MI 24. This experimental model has been widely accepted for studying post-MI LV remodeling and was the basis of our choice to use female rats in our study, in addition to their more frequent availability in our facilities.

The present study showed that the perioperative mortality rate of the MI induction procedure was lower in the previously trained animals than in the non-trained animals. Dayan et al. (3) reported a similar finding, whereas Freimann et al. (6) found similar mortality rates in exercised and non-exercised animals. It is worth noting that in Freimann's study, the infarct sizes were smaller than those found in our study. It is possible that the non-trained animals with larger infarct sizes were the ones that did not survive the MI induction procedure, which could account for the lack of a difference in infarct size between trained and non-trained surviving animals. In contrast, smaller infarct sizes resulting from the MI induction procedure probably did not challenge the myocardium enough to counteract the cardioprotection of training.

The benefits of training, evidenced here by the fact that despite similar infarct sizes or the absence of a difference in LVESD and LVEDD between the groups, the trained animals had better ventricular function and less depression of cardiac function than the non-trained animals did, as indicated by a higher SF.

The attenuation of left ventricular concentricity observed in the trained group demonstrates a compensatory morphological mechanism for maintaining cardiac function. This may also induce cardiomyocyte remodeling, leading to better oxygen uptake, substrate oxidation and fatigue resistance.

The NF-κB family consists of transcriptional factors involved in key reactions of the inflammatory immune response as well as apoptosis.

NF-κB components are maintained in the cytoplasm by the inhibitory action of the protein I-κB, which can be activated by many different stimuli, including ROS, cytokines and bacterial lipids, among others. This protein's activation allows NF-κB to translocate into the cell nucleus.

The hypoxia that occurs during MI increases the production of mitochondrial ROS, resulting in lipid peroxidation, which is a strong inflammatory signal that activates NF-κB. TNF-α and IL-1, cytokines that act as transcriptional activators in vascular endothelial cells, also participate in the activation of the NF-κB pathway by regulating many of its regulatory genes and amplifying the immune response 25. However, anti-inflammatory mediators such as IL-10 and IL-13 suppress production of TNF-α and IL-1, in turn suppressing NF-κB activation by preventing the inactivation of I-κBα.

PPARs are ligand-activated transcription factors that also modulate the activity of genes involved in energy metabolism regulation and inflammatory processes 26. PPAR activators protect against increased activation of caspase-3, an important enzyme in the apoptosis cascade 27. Ligand-activated PPAR-α can directly suppress inflammatory reactions by inhibiting IL-1-induced and NF-κB-mediated production of IL-6, inhibiting NF-κB function (27). High doses of a PPAR-α ligand cause marked activation of NF-κB, whereas low (therapeutic) doses decrease NF-κB activation, IL-6 production, and lipid peroxidation.

The cytokine-mediated inflammatory response is particularly exacerbated after MI, which was well represented in our study by high values of TNF-α at the infarction area of both the sedentary and the exercised groups, after 1 week of healing. This cytokine is not normally present in the healthy myocardium, and its role in MI is far more complex than just activation of a cytokine cascade.

In our study, increased inflammation, represented by higher expression of TNF-α that was not correlated with PPAR-α expression, was observed in SI1 rats. In contrast, in EI1 rats, TNF-α was present at much lower levels, and there was a strong positive correlation with PPAR-α at the infarction area, showing an immunomodulatory effect of the exercise on the inflammatory response.

We also noted that NF-κB was highly expressed at the infarction area in animals from the sedentary and exercised groups after 1 week of healing, with no significant difference between the groups.

The strong positive correlation between PPAR-α and NF-κB only in the previously exercised animals suggests that PPAR-α interacts with TNF-α and NF-κB, which is involved in the development of apoptosis, similar to what occurs in Chagas' disease. In particular, in chagasic myocarditis, PPAR-α ligands exert anti-inflammatory regulation through PPAR-independent mechanisms involving the NF-κB pathway. Treatment with PPAR-α and PPAR-γ ligands drives macrophages toward an M2 profile, inhibiting inflammatory mediators. PPAR signaling is specifically involved in switching macrophage polarity to a tissue-repairing phenotype that might ameliorate inflammatory responses in infectious disease 28 and other inflammatory disorders.

Our study indicates that exercise also seems to be involved in activation of PPAR-α signaling, contributing to decrease the inflammation caused by MI, even in the non-affected myocardium. In the sedentary group, a negative correlation between TNF-α and NF-κB demonstrated such a lack of NF-κB action on TNF-α inhibition. In our study, previous exercise prevented this negative interaction, apparently mediated by activation of PPAR-α. In terms of the long-term outcome, 4 weeks after MI, the activation of PPAR-α remained increased only in previously exercised rats. Furthermore, exercised infarcted animals exhibited higher inflammation levels four weeks after MI. This study also shows that it is important to look for correlations among PPAR-α, TNF-α and NF-κB. Despite the absence of differences in the levels of TNF-α after MI, the correlations are absolutely different.

Taken together, these results suggest that exercise training has the ability to modulate the inflammatory response triggered by MI and to induce a PPAR-α response relative to a lack of training. Coexistence of this response with improvement of post-MI ventricular function may suggest that PPAR-α activation is involved in exercise-dependent attenuation of the myocardial damage. The mechanism possibly involves apoptosis of myocardial cells, which was much more frequent in the sedentary group in the current study. NF-κB, TNF-α and PPAR-α interaction, which was increased in the exercised group, may explain the inhibition of apoptosis.

In conclusion, this study shows that MI in exercise-trained animals is accompanied by substantial changes due to the response of metabolic modulators, inflammatory markers and rates of apoptosis compared with outcomes in non-trained animals. These changes are related to inhibition of TNF-α due to the presence of PPAR-α and NF-κB in favorable metabolic adaptive responses, inhibiting apoptosis and contributing to the improvement of post-MI ventricular performance.

## ACKNOWLEDGMENTS

We thank Mrs. Joyce T. Kawakami for the tissue collection and Mrs. Renata N. Ikegami and Ms. Suely A. P. Palomino, biologists from the Cardiac Pathology Laboratory, for help with the immunohistochemistry and TUNEL techniques. This work was supported by Fundação de Amparo à Pesquisa do Estado de São Paulo (FAPESP) (grant number 06/50489-2) and the Zerbini Foundation.

## Figures and Tables

**Table 1:** Echocardiographic data (mean±SD).

**Group**	**SI4**	**EI4**	*p*	**SSh**	**ESh**	*p*
**MI Size (%)**	34.91±17.47	38.93±13.77	NS	-	-	-
**LVESD**	0.67±0.18	0.77±0.09	NS	0.47±0.06	0.50±0.04	NS
**LVEDD**	0.90±0.14	0.94±0.08	NS	0.76±0.09	0.78±0.04	NS
**SF (%)**	24.13±11.16	35.80±7.80	<0.001	67.95±1.90	69.14±3.26	NS
**E/A Wave**	4.47±1.33	5.50±1.02	NS	2.62±0.91	2.85±1.36	NS

LVESD: Left Ventricular End-Systolic Diameter; LVEDD: Left Ventricular End-Diastolic Diameter; SF: Shortening Fraction.

**Table 2:** Mean±SD of the positive percentage areas for PPAR-α, TNF-α and NF-κB in non-infarcted and infarcted regions.

	**Non-infarcted Region**			**Infarcted Region**		
	**NF-κB**	**PPAR-α**	**TNF-α**	**NF-κB**	**PPAR-α**	**TNF-α**
**SI1**	3.39±0.79	1.45±0.73	2.71±0.99	14.77±5.78	3.06±1.14	9.59±3.82
**EI1**	2.77±0.82	0.91±0.54	0.65±0.22	17.24±16.61	4.83±2.38	4.09±0.92
**SI4**	0.49±0.33	0.53±0.26	1.45±0.52	3.51±2.3	1.59±0.90	4.40±1.58
**EI4**	1.11±0.74	1.36±1.57	2.48±2.40	4.93±2.29	2.88±0.90	5.20±3.20
**SSh**	0.81±0.43	0.71±0.46	1.67±0.74	-	-	-
**ESh**	0.78±0.44	0.87±0.57	2.64±1.40	-	-	-
